# Clustering Properties of Neuronal Ryanodine Receptor 2 and Remodeling in the APP/PS1 Mouse Model of Alzheimer's Disease

**DOI:** 10.1111/apha.70264

**Published:** 2026-06-05

**Authors:** Michelle L. Munro, Ruben Vergara Silva, Shane M. Ohline, Ei Phyo Khaing, Joan A. Chan, Mohamed F. Ibrahim, Tausi F. Tausi, Wickliffe C. Abraham, Peter P. Jones

**Affiliations:** ^1^ Department of Physiology, Faculty of Biomedical Sciences University of Otago Dunedin New Zealand; ^2^ HeartOtago University of Otago Dunedin New Zealand; ^3^ Brain Health Research Centre University of Otago Dunedin New Zealand; ^4^ Department of Psychology University of Otago Dunedin New Zealand

**Keywords:** Alzheimer's disease, CA1 hippocampal neurons, calcium channels, dSTORM, RyR2, super‐resolution imaging

## Abstract

**Aim:**

The ryanodine receptor (RyR2) is an intracellular Ca^2+^ release channel which mediates numerous cellular functions across different tissues. Dysregulation of RyR2 channel activity leads to pathological Ca^2+^ release, which often underlies disrupted cellular signaling in disease states. In the heart, RyR2 channels forms discrete clusters and calcium release units (CRUs) which control channel activity. These structures demonstrate nanoscale remodeling in disease states associated with pathological Ca^2+^ release activity in the heart. Hence, these nanoscale structures are critical in regulating Ca^2+^ release in health and disease. RyR2 is also expressed in brain; however, whether analogous clusters and CRUs form in neurons remains unexplored.

**Methods:**

Using super‐resolution imaging, we assessed RyR2 organization in CA1 pyramidal neurons of wild‐type mice. Furthermore, we used the APP/PS1 mouse model of Alzheimer's disease (AD) to assess whether there is nanoscale remodeling of RyR2 in a setting associated with pathological Ca^2+^ release in neurons.

**Results:**

Here, we provide the first identification and detailed characterization of RyR2 clusters in central nervous system neurons, which are comparable to those reported in the heart. Moreover, we observed a decrease in RyR2 cluster size and reduced CRU organization in AD mice at an age associated with high plaque burden and cognitive deficits. This remodeling is analogous to that reported in pathological states in the heart.

**Conclusion:**

Together, these findings implicate the nanoscale remodeling of RyR2 clusters and CRUs as a novel mechanism underlying Ca^2+^ channel dysregulation and neuronal dysfunction in AD.

## Summary

RyR2 organizes into discrete nanoscale clusters across different tissues. Mirroring the link between cardiac cluster remodeling and heart failure, disruption of these nanoscale patterns in neurons is associated with Alzheimer's disease.

## 1 Introduction

The ryanodine receptor 2 (RyR2) is a Ca^2+^‐sensitive Ca^2+^ release channel which plays a critical role in regulating basic cellular functions across different tissues. In the brain, neuronal RyR2 activity contributes to synaptic transmission, plasticity and intrinsic excitability [1, 2, 3, 4, 5], and is highly implicated in learning and memory [6, 7]. RyR2 localizes to the membrane of the Ca^2+^ store organelle, namely the endoplasmic or sarcoplasmic reticulum (ER or SR, respectively), to mediate intracellular Ca^2+^ release. However, pathological Ca^2+^ release due to RyR2 dysfunction is observed in several disease settings. Alzheimer's disease (AD) is associated with the development of amyloid‐β (Aβ) plaques, neuronal dysfunction and impaired learning and memory. Pathological Ca^2+^ release has been implicated in these hallmarks of AD [8, 9, 10, 11], which is largely linked to altered neuronal excitability due to somatic RyR2 dysfunction [5]. This highlights that RyR2 plays an important role in healthy neuronal function, while dysregulation of RyR2 is implicated in neuronal pathogenesis. However, the mechanisms underlying neuronal RyR2 dysfunction remain poorly understood.

To date, RyR2 has predominantly been studied in the heart, where one mechanism of regulation is the physical organization of channels. We and others have provided extensive evidence that RyR2 channels form discrete clusters in cardiomyocytes [12, 13, 14, 15], and that the nanoscale arrangement of these clusters is tightly associated with Ca^2+^ release properties [16, 17, 18]; for reviews see [19, 20]. Remodeling of these clusters is observed in cardiac diseases associated with pathological Ca^2+^ release, including heart failure [21, 22]. This RyR2 dysfunction results in increased cardiomyocyte excitability and arrhythmogenesis. As RyR2 are Ca^2+^‐sensitive channels, they have the potential to be activated by Ca^2+^ released from neighboring clusters. Therefore, clusters within sufficiently small distances of each other can be considered as functionally coupled. These are referred to as Ca^2+^ release units (CRUs; also called “super‐clusters”), with remodeling of cardiac CRU also associated with pathological Ca^2+^ release in the heart [23]. Considering the similarities in RyR2 dysfunction observed in heart failure and AD which result in increased cellular excitability, it stands to reason that a shared mechanism could underlie pathological Ca^2+^ release in both diseases. However, to date, there is no knowledge on the detailed arrangement of RyR2 channels within central nervous system neurons or whether they form clusters akin to those found in the heart. Without this fundamental knowledge, it also remains unknown if changes in the RyR2 nanoscale arrangement are associated with neuronal diseases linked to RyR2 dysfunction.

In the current study, we used in situ super‐resolution imaging (direct stochastic optical reconstruction microscopy; dSTORM) to investigate whether RyR2 channels in neurons assemble into clusters, analogous to those found in the heart. As the hippocampus is widely implicated in AD pathogenesis, with RyR2 dysfunction identified within the CA1 neurons [9, 11], this was the primary region of interest in this study. We identified, for the first time, that RyR2 channels form clusters in neurons within the adult brain. Detailed characterization revealed that these neuronal clusters are fundamentally like those described in cardiomyocytes. We then explored whether clusters demonstrate nanoscale remodeling in a mouse model of AD, a disease where pathological Ca^2+^ release through somatic RyR2 contributes to the phenotype. We found that RyR2 clusters were smaller in CA1 neurons of AD mice, with an overall loss of channels from CRUs compared to control. This remodeling is analogous to that reported in heart failure. Combined, these findings reveal novel insights into RyR2 organization in neurons, in both healthy and pathological states, suggesting a shared mechanism of RyR2 regulation, which is associated with pathological Ca^2+^ release in different tissues.

## 2 Results

### 2.1 RyR2 Channels Form Nanoscale Clusters in CA1 Hippocampal Neurons

**FIGURE 1:**
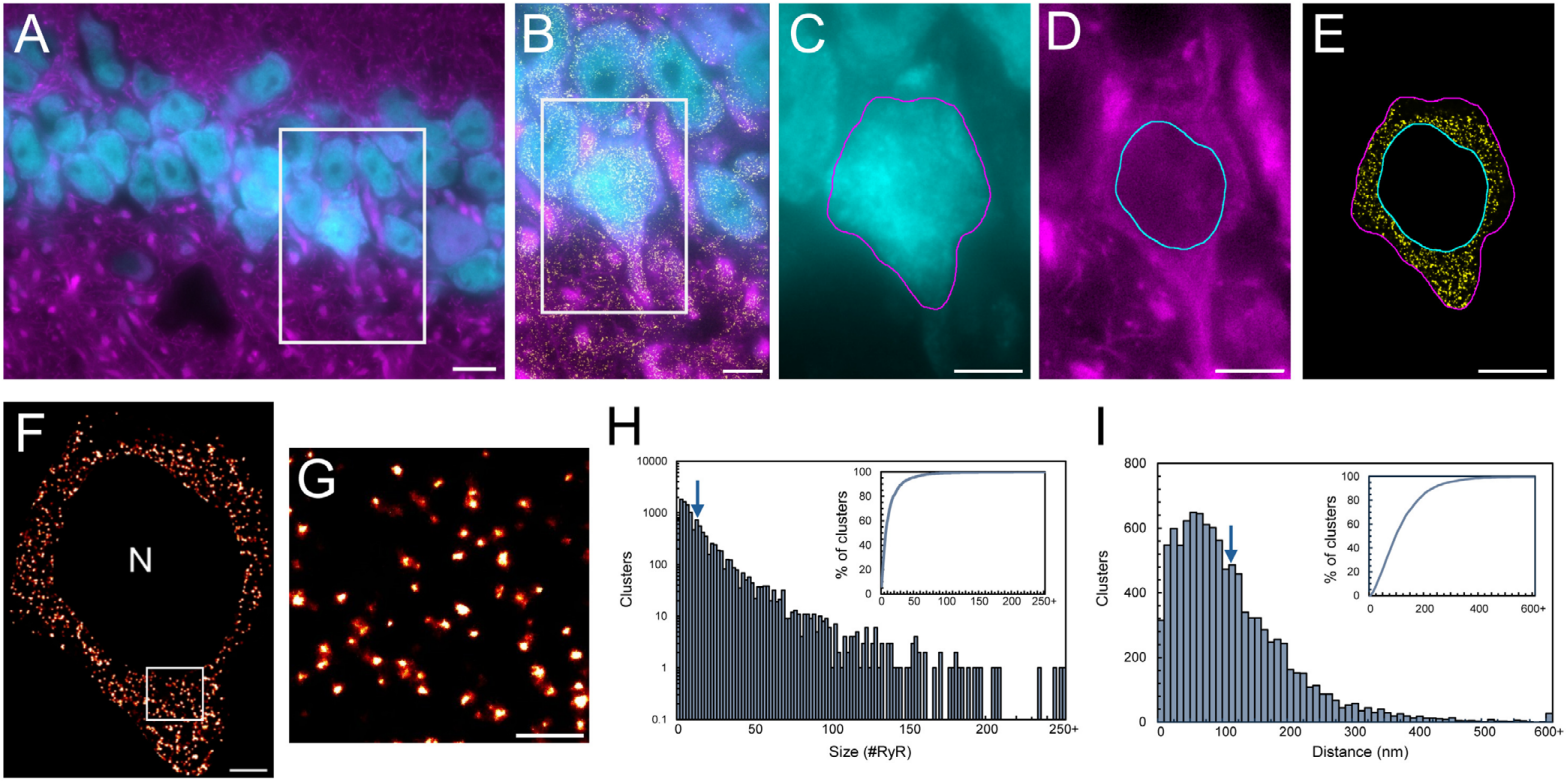
RyR2 channels form clusters in the soma of hippocampal CA1 neurons. (A) Widefield NeuN (cyan) and MAP2 (magenta) labelling in the 9‐month‐old mouse hippocampal CA1 region. Scale bar: 10 μm. (B) Magnified view of the region of interest (ROI; white box in panel A) visualizing nanoscale RyR2 clusters (yellow). Scale bar: 5 μm. (C–E) Segmentation of the soma of a CA1 neuron by determining the boundaries of the neuronal soma (panels C, E magenta line) and nucleus (panels D and E cyan line). Scale bar: 5 μm. (F) Rendered dSTORM image of somatic RyR2 clusters; nuclear region excluded (‘N’). Scale bar: 2 μm. (G) Magnified view of ROI in panel F, demonstrating the identification of discrete RyR2 clusters. Scale bar: 0.5 μm. (H) Frequency histogram of individual cluster sizes, mean size indicated by arrow. Inset shows the cumulative percentage as a function of cluster size. (I) Frequency histogram of inter‐cluster edge‐to‐edge nearest neighbor distance (NND) with mean distance indicated by arrow. The cumulative percentage of NND values is displayed in the inset. Data collected from *n* = 36 cells, *N* = 5 animals.

The nanoscale organization of somatic RyR2 channels was investigated within hippocampal CA1 neurons in the adult mouse brain, using NeuN and MAP2 staining to identify neuronal structure and allow for selective segmentation of the soma (Figure 1A–E). Rendered dSTORM images revealed the clear formation of discrete RyR2 clusters in the somata of CA1 neurons (Figure 1F,G). These clusters ranged in size, from apparent single channels through to enlarged clusters, with a near‐exponential distribution (Figure 1H). The mean area was 13 420 ± 499 nm^2^, corresponding to a cluster size of 14.9 ± 0.6 RyR2 channels (assuming isotropic packing [24]). Furthermore, ~50% of clusters contained 6 or fewer channels (Figure 1H, inset), demonstrating an overall small size of neuronal somatic RyR2 clusters. These clusters were observed throughout the soma, with a mean distance separating nearest neighboring clusters (NND) of 118.8 ± 3.8 nm (Figure 1I).

### 2.2 CRU Formation by Neuronal RyR2 Clusters

**FIGURE 2:**
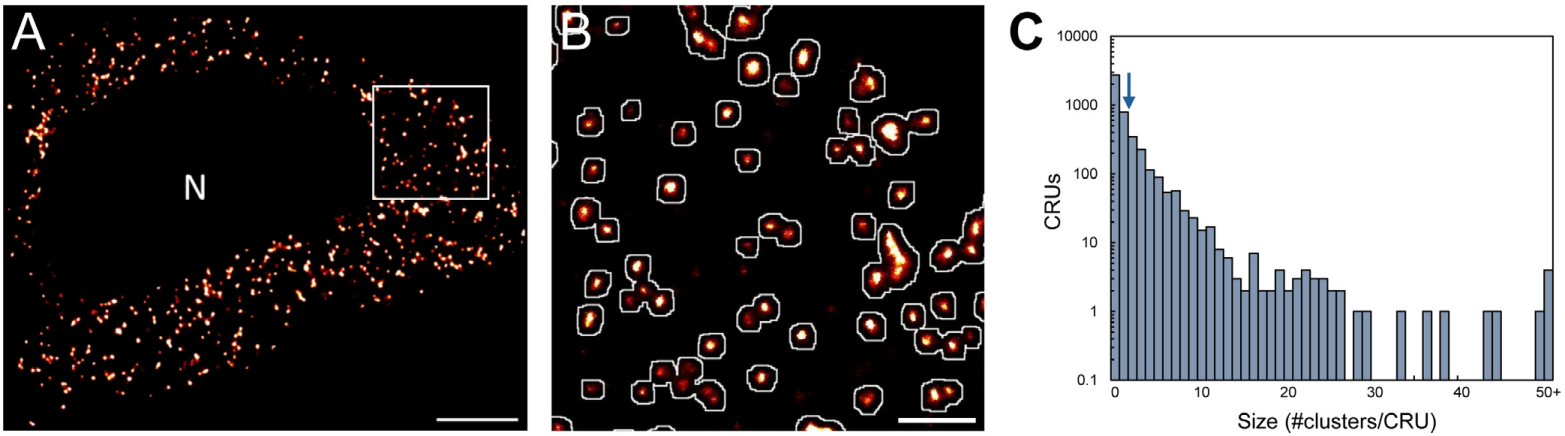
Formation of CRUs in hippocampal CA1 neuronal soma. (A) Rendered dSTORM image of somatic RyR2 clusters selectively cropped from a CA1 neuron in the wildtype mouse hippocampus; nuclear region excluded from analysis indicated by “N”. Scale bar: 2 μm. (B) Magnified view of region of interest in panel A (white box), demonstrating the definition and identification of Ca^2+^ release units (CRUs; white outlines) overlaid on super‐resolved somatic RyR2 clusters. Scale bar: 0.5 μm. (C) Frequency histogram of CRU composition (on a logarithmic scale) demonstrating the distribution of CRU sizes, with a mean of 2.4 individual RyR2 clusters per CRU (indicated by arrow). Data collected from *n* = 36 cells, *N* = 5 animals.

Due to the potential cooperative nature of Ca^2+^ signaling between RyR2 clusters within sufficiently small neighboring distances, we next explored whether neuronal RyR2 clusters demonstrate potential CRU formation, with clusters located < 150 nm of each other considered to be grouped into a CRU (Figure 2A,B). Euclidean edge‐to‐edge distance analysis revealed that ~60% of clusters containing two or more RyR2 channels were “isolated” from neighboring clusters, such that these CRUs contained a single RyR2 cluster (Figure 2C). The remaining neuronal clusters were located within a sufficiently small distance from neighboring clusters to form multi‐cluster CRUs. These multi‐cluster CRUs predominantly contained 2–8 individual RyR2 clusters, with a mean of 2.4 ± 0.1 clusters/CRU (Figure 2C).

### 2.3 RyR2 Expression Is Reduced in the AD Mouse Hippocampus

**FIGURE 3:**
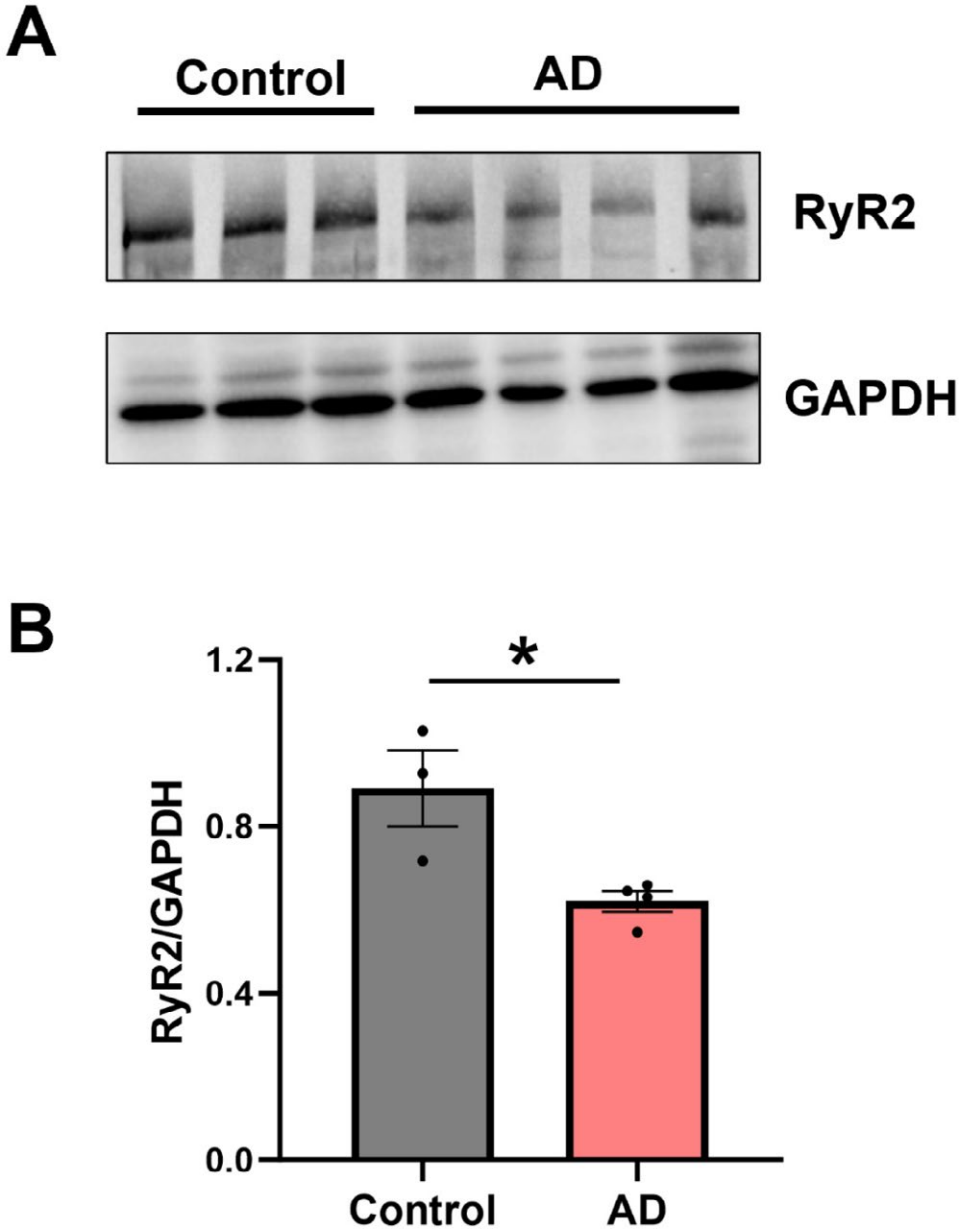
RyR2 expression is reduced in the AD mouse hippocampus. (A) Representative western blots showing total RyR2 and GAPDH expression in hippocampal tissue from control and AD mouse brains. (B) Densitometry analysis of RyR2 expression normalized to GAPDH reveals reduced expression in AD hippocampi. Data displayed shown as mean ± SEM; *N* = 3 control, 4 AD animals. Data analyzed using Student's unpaired *t*‐test; *p = 0.022. Samples run as technical triplicates.

Altered RyR2 expression is observed in cardiac pathologies associated with increased pathological Ca^2+^ release activity [25, 26], which has also been implicated in neuronal dysfunction in AD [10, 11, 27]. Therefore, we aimed to establish whether hippocampal RyR2 expression is also altered in our 9 month old APP/PS1 mouse model of AD. Western blotting confirmed the expression of RyR2 in the mouse hippocampus, with positive bands present in tissue samples from control wildtype (control) and AD mouse brains (Figure 3A). Densitometric analysis revealed a ~30% reduction in hippocampal RyR2 expression in the AD brain (0.62 ± 0.03 A.U.) compared to control (0.89 ± 0.09 A.U.), normalized to GAPDH (Figure 3B; *p* = 0.02).

### 2.4 Remodeling of Somatic RyR2 Clusters in AD Mouse CA1 Neurons

**FIGURE 4:**
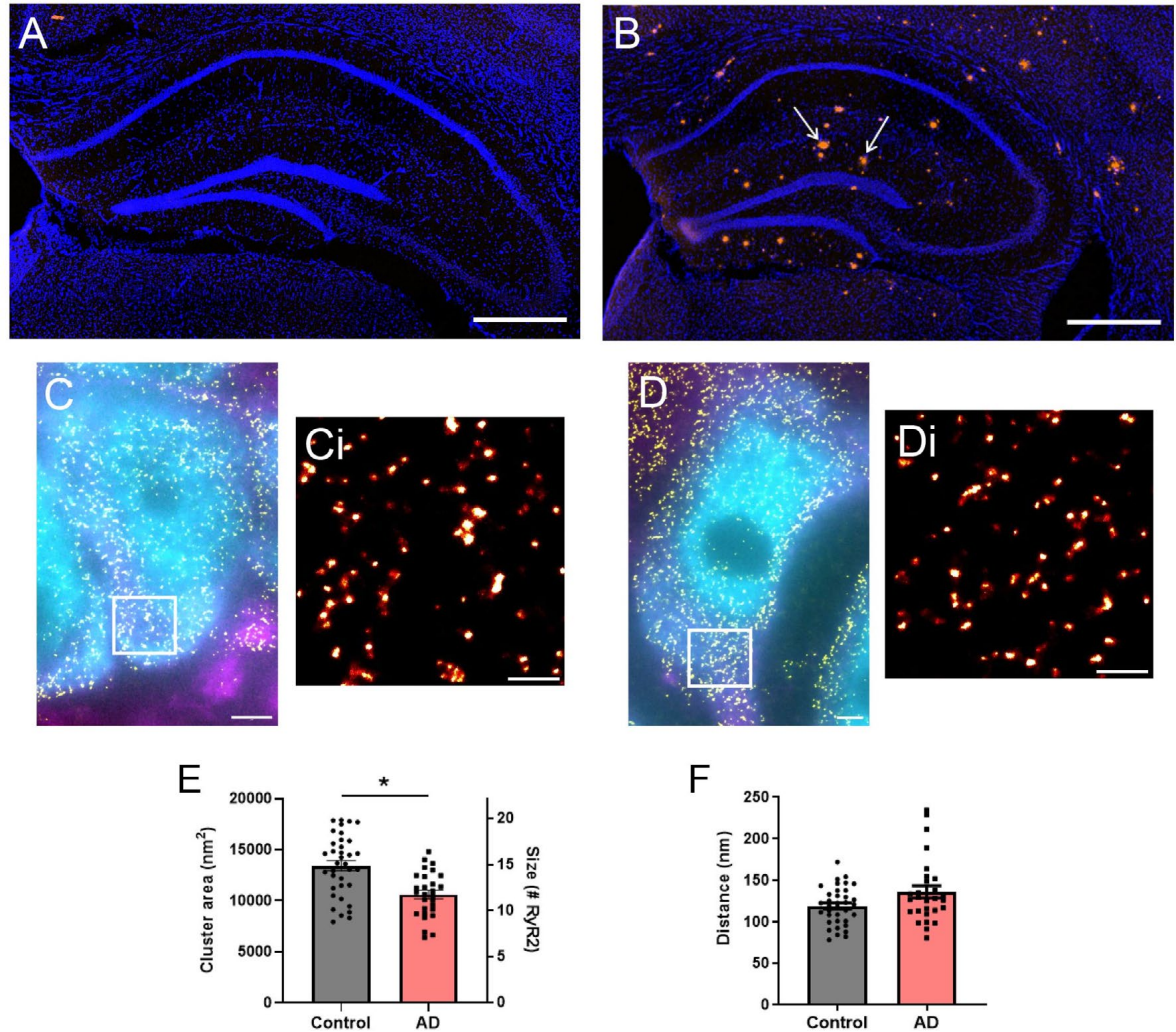
Neuronal RyR2 clusters are altered in a mouse model of AD. DAPI staining (blue) and amyloid‐β plaque deposition (Congo red staining; arrows) in the hippocampus of 9‐month‐old (A) control and (B) AD mice. Scale bar: 500 μm. Widefield NeuN (cyan) and MAP2 (magenta) labelling overlaid with super‐resolved RyR2 clusters (yellow) in the hippocampal CA1 region of (C) control and (D) AD mice. Scale bar: 2 μm. Magnified view of the region of interest (ROI; white box) in panels C and D, visualizing RyR2 clusters in (Ci) control and (Di) AD mice. Scale bar: 0.5 μm. Analysis of (E) RyR2 cluster size and (F) inter‐cluster distance (nearest neighbor, NND) in control and AD mice. Data displayed as mean ± SEM; *n* = 36 (control), 28 (AD) cells per group collected, *N* = 5 (control), 5 (AD) animals. Data analyzed using a Linear Mixed Effects model. RyR2 cluster size: *p = 0.01; inter‐cluster distance: *p* = 0.32.

As morphological changes in RyR2 clusters and CRUs are associated with altered RyR2 function in the heart [21, 23, 25], we assessed whether analogous remodeling is also observed in settings associated with pathological neuronal Ca^2+^ release, using AD mice. At 9 months of age, these AD mice demonstrate cognitive impairment [28, 29] and significant plaque deposition (Congo red staining) in the hippocampus, which is virtually absent in control mice (Figure 4A,B). Analysis of dSTORM images from AD and control mice (Figure 4C,D) identified significant nanoscale remodeling of RyR2 clusters in the somata of CA1 neurons in AD mice. This included a smaller mean cluster size compared to control mice (AD 10 600 ± 419 nm^2^, corresponding to 11.8 ± 0.5 RyR2 channels; *p* = 0.01; Figure 4E), indicating a loss of channels from somatic clusters in AD. Given the evidence that inter‐cluster remodeling is observed in cardiac pathologies, we also assessed this parameter in the AD mice. This revealed that, unlike in cardiac cells, the NND was not changed within AD neurons (135.8 ± 7.4 nm; *p* = 0.32; Figure 4F).

### 2.5 Neuronal RyR2 Clusters and Channels Are Lost From CRUs in the AD Mouse

**FIGURE 5:**
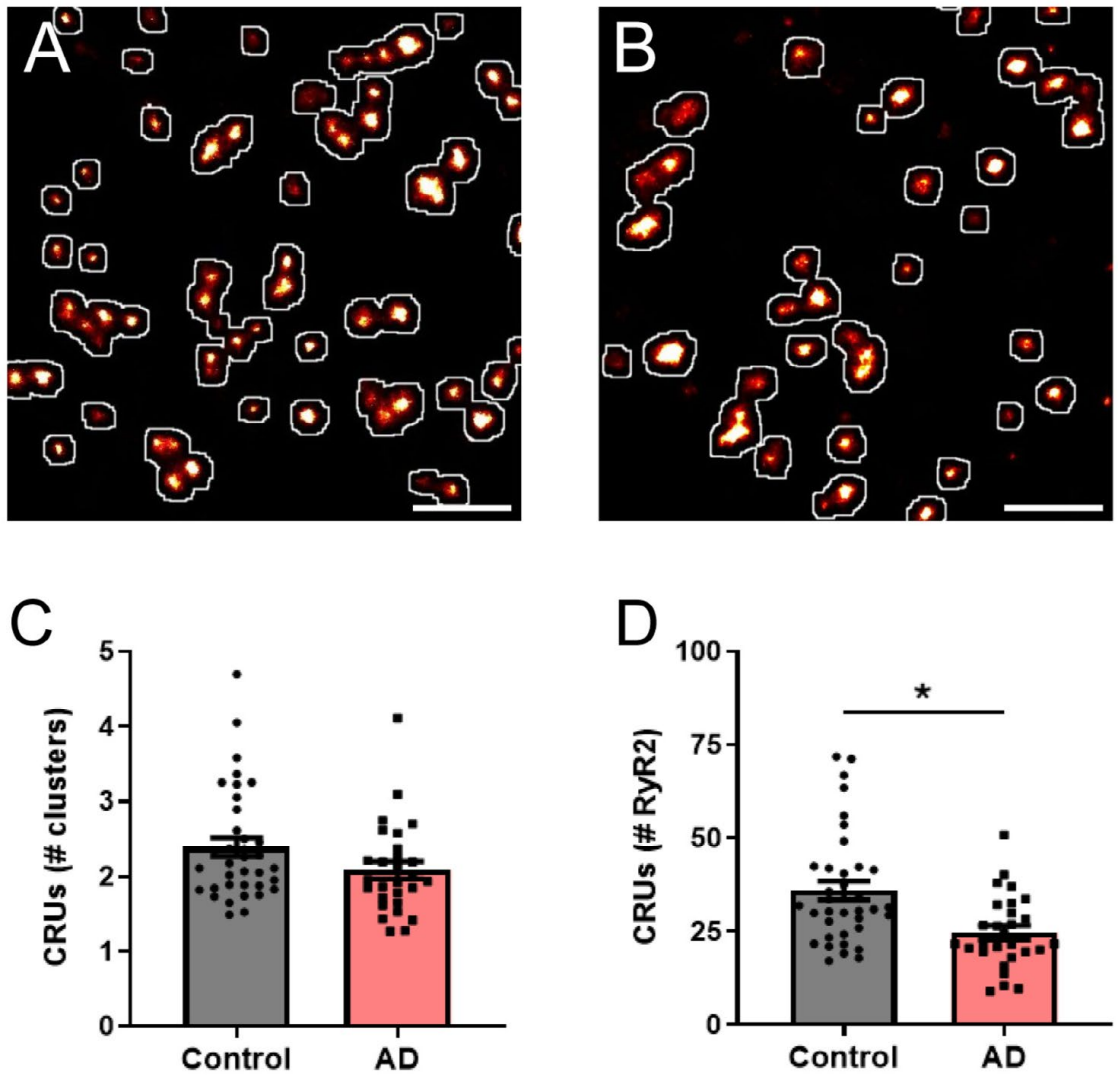
RyR2 clusters and channels are lost from CRUs in AD. Rendered dSTORM image of RyR2 labelling demonstrating the identification of CRU formation (white outlines) in hippocampal CA1 neurons from (A) control and (B) AD mice. Scale bar: 0.5 μm. Image analysis reveals the characteristics of somatic neuronal CRUs, including the mean (C) number of individual RyR2 clusters contained within a CRU, and (D) calculated number of RyR2 channels per CRU in control and AD mice. Data displayed as mean ± SEM; *n* = 36 (control), 28 (AD) cells from *N* = 5 (control), 5 (AD) animals. Data analyzed using a Linear Mixed Effects model. Clusters/CRU: *p* = 0.34; RyR2/CRU: *p = 0.05.

The arrangement of RyR2 clusters into CRUs has been previously shown to be altered in cardiac pathologies in which RyR2 activity is dysregulated [21, 23]. Therefore, we assessed whether CRU remodeling also occurs in our mouse model of AD. Image processing confirmed CRU formation occurs in the soma of CA1 neurons in both control and AD mice (Figure 5A,B). Analysis revealed that there was no change in the mean number of clusters contained within a CRU in AD mice, compared to control mice (AD 2.1 ± 0.1 clusters/CRU; *p* = 0.34; Figure 5C). Considering the significant reduction in RyR2 cluster size identified in the AD mice, we determined the impact on CRU composition by calculating the number of RyR2 channels per CRU. This showed a ~31% loss of RyR2 channels from CRUs in AD mice compared to control. Mean values were: control 36.0 ± 2.5 RyR2/CRU, AD 24.7 ± 1.8 RyR2/CRU (*p* = 0.050; Figure 5D).

Combined, these findings reveal the formation of discrete neuronal RyR2 clusters in the adult brain, which demonstrate significant nanoscale remodeling in a mouse model of AD associated with pathological Ca^2+^ release and neuronal dysfunction.

## 3 Discussion

This study provides the first identification and detailed characterization of RyR2 clusters in the brain. Furthermore, these clusters are altered in a mouse model of AD. While nanoscale RyR2 remodeling is associated with channel dysfunction in the heart [21, 22, 23], to our knowledge, this mechanism has not been previously explored in the brain. Our findings provide a completely new mechanism through which neuronal RyR2 might be regulated in health and disease, and offer new insights into the role of RyR2 in AD.

It is well established that RyR2 forms discrete clusters in cardiomyocytes, with this structural arrangement tightly linked to channel activity in the heart. The organization of cardiac RyR2 influences channel activation, with the arrangement into clusters proposed to regulate physiological Ca^2+^ release [30, 31]. However, it is unclear whether analogous structures form and remodel in the brain. In neurons, somatic RyR2 channel activity influences afterhyperpolarization to modulate intrinsic excitability [5]. We identified that RyR2 channels also form discrete clusters within the somata of hippocampal CA1 neurons. These somatic clusters demonstrate fundamental properties which are similar to those reported in cardiomyocytes, with a mean size of ~9–19 RyR2 channels in cardiac clusters [15, 21, 25, 32]. The identification of somatic RyR2 clusters provides a novel mechanism by which RyR2 activity and neuronal intrinsic excitability may be regulated. RyR2 Ca^2+^ release also facilitates neurotransmitter release [1] and has been implicated in synaptic plasticity within the hippocampus [2, 3, 33]; however, recent studies suggest that RyR2 is absent from dendritic spines of CA1 hippocampal neurons [34, 35]. Given the complex architecture of the dendritic arbour within the hippocampus, the identification and segmentation of individual neuronal processes for RyR2 cluster analysis would be technically challenging, and therefore was not investigated within this study.

As RyR2 channels are Ca^2+^‐sensitive, clusters have the potential to be functionally coupled through inter‐cluster Ca^2+^ propagation to regulate coordinated, cell‐wide Ca^2+^ release activity. In cardiac tissue, modeling suggests that clusters separated by sufficiently small distances (< 150 nm) demonstrate this property, giving rise to the formation of CRUs [23]. Cardiac clusters demonstrate inter‐cluster distances that facilitate CRU formation [13, 15], as do the somatic RyR2 clusters we identified. Our study confirms CRU formation in the somata of CA1 neurons, with an average of ~2.4 individual clusters per CRU, which is comparable to the 2.1–3.7 clusters reported for cardiac CRUs [13, 15, 21]. However, it must be noted that whilst 150 nm is commonly used in cardiac studies, it is largely based on modeling rather than empirical data. Given the differences in somatic and cardiac myocyte ultrastructure, whether this threshold is directly translatable remains to be determined.

Altered RyR2 activity has been identified as contributing to neuronal dysfunction in disease states. Reduced RyR2 expression has been reported in failing human hearts [25], but is not observed in all pathologies associated with cardiac RyR2 hyperactivity [26]. Similarly, there is disparity in reports of altered RyR2 expression in AD [9, 36, 37, 38], which may reflect differences in disease progression or animal models. Our 9 month old APP/PS1 mouse model of AD demonstrates a significant reduction in hippocampal RyR2 expression, which is in agreement with findings from AD patients [39].

In AD, dysfunction of somatic RyR2 has been associated with altered membrane properties in hippocampal neurons, contributing to learning and memory impairments, while inhibition of pathological RyR2 Ca^2+^ release can prevent the progression of AD‐like symptoms [11, 27, 40]. Furthermore, it has been proposed that pathological Ca^2+^ release contributes to the development of hallmark Aβ plaques in the AD brain [10, 36]. Our findings reveal a reduction in neuronal RyR2 cluster size in the AD mouse brain at an age associated with significant Aβ plaque burden and cognitive deficits [28, 29]. This cluster remodeling is analogous to that observed in cardiac settings associated with pathological RyR2 Ca^2+^ release, including heart failure [21, 22]. As smaller clusters contain fewer channels, stabilizing channel‐channel interactions are lost [41, 42], resulting in increased channel open probability and enhanced pathological Ca^2+^ release [18, 43, 44]. Together, this indicates that reduced cluster size is a common mechanism associated with pathological Ca^2+^ release by RyR2 in both the heart and the brain. Given the progressive nature of AD, determining the timing of RyR2 cluster remodeling in relation to AD symptomology would be of future research interest and help determine if the changes observed are causal of disease progression or a consequence of the disease.

The proximity of RyR2 clusters relative to each other influences Ca^2+^ release dynamics. In failing human hearts, an increased distance between neighboring clusters has been reported [25], but this was not observed in the AD brain. Unsurprisingly, this resulted in no change in the number of clusters per CRU. However, the reduction in size of individual clusters within a CRU resulted in a ~31% loss of RyR2 channels from CRUs in AD. A similar loss of RyR2 channels is also observed in heart failure CRUs and is associated with increased pathological Ca^2+^ release [21]. Overall, our findings of altered cluster and CRU organization are in agreement with the observed decrease in RyR2 expression in the AD mice; however, it should be acknowledged that the latter finding was from whole hippocampal tissue and not limited to CA1 pyramidal neurons.

## 4 Conclusion and Significance

Our findings indicate that RyR2 clustering and CRU formation are a common property of the channel and likely represent a fundamental mechanism of regulating RyR2 activity to influence cellular excitability in health and disease. Previous studies targeting RyR2 through direct channel inhibition demonstrate the potential to prevent AD‐like symptom development. Our study indicates that targeting RyR2 organization as an alternative strategy might also be beneficial to prevent pathological Ca^2+^ release and neuronal dysfunction in AD.

## 5 Materials and Methods

### 5.1 Animals and Tissue Samples

Nine‐month‐old female mice were utilized for this study. The AD mouse model comprised hemizygous double transgenic mice expressing the Swedish mutation to amyloid precursor protein (APPswe) and the exon 9 deletion in presenilin 1 (PS1ΔE9) of the human APP695 protein on a C57BL/6J background (APP/PS1; AD mice) [45]. Wildtype littermates were used as controls. All animal research was approved by University of Otago Ethics Committee (#AUP.20‐91) and complied with the New Zealand Animal Welfare Act 1999. Mice were anesthetized by intraperitoneal injection of 125 mg/kg sodium pentobarbital, then perfused with phosphate buffered saline (PBS), followed by 4% paraformaldehyde in PBS (PFA) at ~5 mL/min. Brains were extracted and post‐fixed in 4% PFA for 1 h at room temperature before cryoprotection in 30% sucrose. The brains were grossly sectioned, embedded in optimum cutting temperature compound O.C.T (Tissue TEK), then frozen in the vapor phase of liquid nitrogen and stored at −80°C until use.

### 5.2 Immunofluorescence

Cryosections containing the dorsal hippocampus (dSTORM: 8 μm, confocal: 30 μm) were collected onto no. 1.5 glass coverslips coated with 0.05% poly‐l‐lysine. Sections were permeabilised with 1% Triton‐X100 for 10 min before blocking with 10% normal goat serum + 0.3 M glycine in PBS for 1 h. Rabbit anti‐RyR2, guinea pig anti‐NeuN and chicken anti‐MAP2 were incubated overnight at 4°C. Fluorophore‐conjugated secondary antibodies were applied for 2 h at room temperature: goat anti‐rabbit Alexa Fluor 680 or 647, goat anti‐guinea pig Alexa Fluor 555 and goat anti‐chicken Alexa Fluor 488. Antibody dilutions and manufacturer details are provided in Table S1. Coverslips were mounted onto microscope slides with photo‐switching buffer [14].

### 5.3 Confocal and dSTORM Imaging

Confocal microscopy was performed using a Nikon A1+ inverted laser scanning microscope and Nikon NIS Elements software (Nikon, Japan) [14]. Overview images were acquired using a Nikon 20× 0.75 NA objective, with detailed images of CA1 neurons captured with a Nikon 60× oil immersion 1.4 NA objective.

dSTORM imaging was performed using an Olympus IX81 inverted microscope equipped with a 60× oil immersion 1.45 NA TIRF objective using previously described methods [14]. Alexa Fluor 488 and 555 were visualized to obtain widefield images of MAP2 and NeuN labelling, respectively. Images were then corrected for temporal drift and rendered into a greyscale 5 × 5 nm/pixel 16‐bit tiff image, as previously described [46]. Image acquisition, event localization, and greyscale rendering were achieved using previously described custom‐written Python Microscopy Environment software (PyME) (https://bitbucket.org/david_baddeley/python‐microscopy).

### 5.4 Image Analysis

Rendered RyR2 dSTORM images [14, 46] were overlaid with corresponding widefield MAP2 and NeuN images to identify RyR2 clusters localized to the soma of the hippocampal CA1 pyramidal cells. Somatic RyR2 clusters within CA1 neurons were cropped for analysis based on previously established methods using PyME and ImageJ [13, 14, 17]. A binary mask was produced such that the mask contained 75% of the total labelling intensity and was used to determine the RyR2 clustering parameters. Individual regions of interest were assigned to each RyR2 cluster for analysis, excluding particles smaller than a single channel (< 900 nm^2^) [25]. Cluster area and maximum number of channels per cluster were determined based on previous methods [13]. Cluster density was calculated by dividing the total number of clusters per soma by the image area analyzed (excluding the nucleus). The Euclidean edge‐to‐edge distance was measured to determine nearest neighbor distance (NND) and the formation of CRUs by determining the number of individual clusters that were within 150 nm from each other [14, 21, 23].

### 5.5 Western Blotting

Lysates were prepared from isolated mouse hippocampal tissue, separated using SDS‐PAGE and probed for RyR2 and GAPDH, based on previously described methods [47]. Primary antibodies used were: rabbit anti‐RyR2 (Atlas Antibodies, HPA020028; 1:1000) and mouse anti‐GAPDH‐HRP (Abcam, ab9482; 1:5000). Goat anti‐rabbit Alexa Fluor 680 (1:2500 for RyR2) and anti‐mouse IgG peroxidase (1:10 000 for GAPDH) secondary antibodies were incubated for 1 h at room temperature: GAPDH bands were detected by SuperSignal West Pico Plus Chemiluminescent Substrate (ThermoFisher Scientific) and RyR2 bands were detected using epi‐far red 650–675 nm excitation. Blots were imaged using a ChemiDoc MP Imaging system (Bio‐Rad). The band density was analyzed using ImageJ software, with RyR2 expression normalized to GAPDH expression.

### 5.6 Statistical Analysis

Comparison of RyR2 expression was performed using Prism 7 (GraphPad Software, San Diego, USA). Comparison of RyR2 clusters was performed using a Mixed Linear Effects model in MATLAB R2024a (Natick, Massachusetts, USA). For all analysis, a *p*‐value ≤ 0.05 was considered statistically significant. For image analysis, a lowercase *n* represents the number of cells per group, while an uppercase *N* corresponds to the number of animals used. The *n* and *N‐*values, *p‐*values, and statistical analyses performed are reported in the corresponding figure legends.

## Author Contributions

**Wickliffe C. Abraham:** resources, writing – review and editing. **Michelle L. Munro:** methodology, investigation, supervision, visualization, writing – original draft, writing – review and editing, formal analysis, data curation. **Ruben Vergara Silva:** investigation, visualization, writing – review and editing. **Joan A. Chan:** investigation, writing – review and editing. **Mohamed F. Ibrahim:** methodology, writing – review and editing. **Tausi F. Tausi:** writing – review and editing, formal analysis. **Shane M. Ohline:** methodology, investigation, supervision, writing – review and editing. **Peter P. Jones:** conceptualization, methodology, funding acquisition, resources, supervision, writing – original draft, writing – review and editing, formal analysis, data curation, project administration. **Ei Phyo Khaing:** investigation, writing – review and editing.

## Funding

This study was supported by The Health Research Council of New Zealand Grant 20/370 (P.P.J.) and 20/625 (M.L.M.), The Neurological Foundation of New Zealand, supported by Thanksgiving Trust, Mrs. Joy Bollard and Barbara M. Brockie 2011 PGR (P.P.J.) and the Marsden Fund from the Royal Society of New Zealand UOO2009 (M.L.M.).

## Ethics Statement

All animal research was approved by the University of Otago Ethics Committee (#AUP.20‐91) and complied with the New Zealand Animal Welfare Act 1999.

## Conflicts of Interest

The authors declare no conflicts of interest.

## Supporting information


**Table S1:** Antibody details for immunofluorescence.

## Data Availability

All data underlying the study will be made openly available in the Github (github.com) repository.
