# Supplementary material for: Clustering Properties of Neuronal Ryanodine Receptor 2 and Remodeling in the APP/PS1 Mouse Model of Alzheimer's Disease
Source: Acta Physiol (Oxf). 2026 Jun 5;242(7):e70264. doi: 10.1111/apha.70264 (PMC13241622; doi:10.1111/apha.70264)
Supplement: Supplementary file 1 — Table S1: Antibody details for immunofluorescence. [file APHA-242-e70264-s001.docx]

Clustering properties of neuronal ryanodine receptor 2 and remodelling in the APP/PS1 mouse model of Alzheimer’s disease

Michelle L. Munro,^a,b,1^* Ruben Vergara Silva,^a,b,c,1^ Shane M. Ohline,^a,b,c^ Ei Phyo Khaing,^a,b^ Joan A. Chan,^a,b,c^ Mohamed F. Ibrahim,^a,c,2^ Tausi F. Tausi,^a,b^ Wickliffe C. Abraham,^c,d^ Peter P. Jones^a,b,c^*

**Supplementary Materials**

**Supplementary Table S1. Antibody details for immunofluorescence**

| **Target** | **Species** | **Dilution*** | **Manufacturer details** |
| --- | --- | --- | --- |
| MAP2 | Chicken | 1:500 | Abcam; Ab5392 |
| NeuN | Guinea pig | 1:1,000 | Synaptic Systems; 266004 |
| RyR2 | Rabbit | 1:500 | Atlas Antibodies; HPA020028 |
| Anti-chicken Alexa Fluor 488 | Goat | 1:500 | Abcam; Ab150173 |
| Anti-guinea pig Alexa Fluor 555 | Goat | 1:500 | Life Technologies; A21435 |
| Anti-rabbit Alexa Fluor 647 (confocal) | Goat | 1:500 | Life Technologies; A21245 |
| Anti-rabbit, Alexa Fluor 680 (dSTORM) | Goat | 1:200 | Life Technologies; A21109 |

*Antibodies diluted in PBS with 1% bovine serum albumin + 0.05% Triton-X100 + 0.05% NaN_3_
